# EDTA soluble chemical components and the conditioned medium from mobilized dental pulp stem cells contain an inductive microenvironment, promoting cell proliferation, migration, and odontoblastic differentiation

**DOI:** 10.1186/s13287-016-0334-z

**Published:** 2016-05-25

**Authors:** Rei Kawamura, Yuki Hayashi, Hiroshi Murakami, Misako Nakashima

**Affiliations:** Department of Stem Cell Biology and Regenerative Medicine, National Center for Geriatrics and Gerontology, Research Institute, 7-430 Morioka, Obu, Aichi 474-8511 Japan; Department of Gerontology, School of Dentistry, Aichi-Gakuin University, Nagoya, Aichi 464-8651 Japan; Department of Oral Implantology, School of Dentistry, Aichi-Gakuin University, Nagoya, Aichi 464-8651 Japan; Department of Pediatric Dentistry, School of Dentistry, Aichi-Gakuin University, Nagoya, Aichi 464-8651 Japan

**Keywords:** Dental pulp mesenchymal stem cells, Chemical microenvironment, Pulp/dentin regeneration, Migration, Odontoblastic differentiation, Angiogenesis, Cell attachment

## Abstract

**Background:**

The critical challenge in tissue engineering is to establish an optimal combination of stem cells, signaling morphogenetic molecules, and extracellular matrix scaffold/microenvironment. The extracellular matrix components of teeth may be reconstituted as an inductive microenvironment in an ectopic tooth transplantation bioassay. Thus, the isolation and identification of the chemical components of the inductive microenvironment in pulp/dentin regeneration will accelerate progress towards the goal of tissue engineering of the tooth.

**Methods:**

The teeth demineralized in 0.6 M hydrochloric acid were sequentially extracted by 4.0 M guanidine hydrochloride (GdnHCl), pH 7.4, and 0.5 M ethylenediaminetetraacetic acid (EDTA), pH 7.4. The extracted teeth were transplanted into an ectopic site in severe combined immunodeficiency (SCID) mice with mobilized dental pulp stem cells (MDPSCs). The unextracted tooth served as a positive control. Furthermore, the soluble components for the inductive microenvironment, the GdnHCl extracts, or the EDTA extracts together with or without MDPSC conditioned medium (CM) were reconstituted systematically with autoclaved teeth in which the chemical components were completely inactivated and only the physical microenvironment was preserved. Their pulp/dentin regenerative potential and angiogenic potential were compared 28 days after ectopic tooth transplantation by histomorphometry and real-time RT-PCR analysis.

**Results:**

Expression of an odontoblastic marker, *enamelysin*, and a pulp marker, thyrotropin-releasing hormone degrading enzyme (*TRH-DE*), was lower, and expression of a periodontal cell marker, anti-asporin/periodontal ligament-associated protein 1 (PLAP-1), was higher in the transplant of the EDTA-extracted teeth compared with the GdnHCl-extracted teeth. The autoclaved teeth reconstituted with the GdnHCl extracts or the EDTA extracts have weak regenerative potential and minimal angiogenic potential, and the CM significantly increased this potential. Combinatorial effects of the EDTA extracts and the CM on pulp/dentin regeneration were demonstrated in vivo, consistent with their in-vitro effects on enhanced proliferation, migration, and odontoblastic differentiation*.*

**Conclusions:**

The EDTA-extracted teeth demonstrated significantly lower pulp/dentin regenerative potential compared with the GdnHCl-extracted teeth. The EDTA soluble chemical components when reconstituted with the physical structure of autoclaved teeth serve as an inductive microenvironment for pulp/dentin regeneration, promoting cell proliferation, migration, and odontoblastic differentiation.

**Electronic supplementary material:**

The online version of this article (doi:10.1186/s13287-016-0334-z) contains supplementary material, which is available to authorized users.

## Background

The human body has biological mechanisms for regeneration of tissue damage by recapitulation as part of embryonic development and morphogenesis. The critical challenge in tissue engineering and regeneration is to establish an optimal combination of the triad of stem cells, signaling molecules, and extracellular matrix scaffold/microenvironment for pulp/dentin regeneration. Mesenchymal stem cells (MSCs) exhibit trophic properties by bioactive factors, which directly trigger intracellular mechanisms of damaged cells or indirectly enhance release of the functionally active signals by adjacent cells [1, 2]. The cytokines, inflammatory mediators, extracellular matrix components, and antimicrobial proteins released by MSCs generate an appropriate microenvironment for tissue repair [3]. Our work demonstrated that quantitatively similar tissues were regenerated after transplantation of pulp, bone marrow, and adipose tissue-derived stem cells in the rat brain ischemic model, the mouse hind-limb ischemic model, and the dog pulpitis model [4, 5]. Furthermore, conditioned medium (CM) from pulp induced a higher volume of regenerated pulp tissue compared with CM from bone marrow and adipose tissue-derived stem cells in the ectopic tooth transplantation model, due to potent trophic factors which enhance migration and angiogenesis. The regenerated tissues, however, were quantitatively similar among the three distinct transplants of CM [6]. These reports indicate that the regenerated tissue is independent of stem cell origin. A multitude of cell responses are governed by complex chemical and physical cues from the surrounding microenvironment encompassing different length scales, from nano to micro [7, 8], although the mechanisms of these are poorly understood. The fate of progenitor/stem cells is influenced by both soluble and insoluble factors. Many intrinsic soluble factors, however, form matrix-associated insoluble complexes and are regulated in their release and activation from the surrounding microenvironment [9].

Engineered microenvironments from the construction of synthetic extracellular matrix-mimetic systems for programmed stem cell response are becoming increasingly a productive approach to determine structural and functional complexity of the physiological environment in vivo around the cells. Tooth loss greatly affects quality of life. Developing tissue engineering for natural tooth including dentin/pulp complex and periodontal tissue, however, potentially overcomes limitations of conventional replacement with dentures or synthetic artificial dental implants. Our previous studies [4, 6] and other studies [10, 11] suggest the dentin matrix itself to be an inductive microenvironment for dentin/pulp regeneration. The chemical factors contained in dentin matrix have not been identified. Therefore, in this investigation, the pulp/dentin regenerative potential was compared in teeth extracted sequentially with hydrochloric acid (HCl), guanidine hydrochloride (GdnHCl), and ethylenediaminetetraacetic acid (EDTA) by an ectopic tooth transplantation assay harnessing mobilized dental pulp stem cells (MDPSCs). In addition, the optimal extract from the teeth was examined for pulp/dentin regeneration after reconstitution with the autoclaved teeth.

## Methods

All animal experiments were conducted using the strict guidelines of the Animal Protocol Committees (authorization number: AGUD156) and DNA Safety Programs at both the National Center for Geriatrics and Gerontology, Research Institute and Aichi-Gakuin University.

### Cell isolation and preparation of CM

The primary pulp cells were isolated from the pulp tissue of porcine premolar teeth with slight modification as described previously [12]. The isolated cells were plated at colony-forming density (≤1 × 10^4^ cells/ml) on 35 mm dishes (BD Biosciences, San Jose, CA, USA) in Dulbecco’s modified Eagle’s medium (DMEM) (Sigma Aldrich, St. Louis, MO, USA) supplemented with 10 % fetal bovine serum (FBS) (Life Technologies, Carlsbad, CA, USA). These colony-derived DPSCs at the second passage of culture at 1.5 × 10^4^ cells/100 μl in DMEM were added to the upper chambers of Costar Transwell® (Corning, Lowell, MA, USA) which was chemically pretreated to prevent cell attachment. DMEM supplemented with 10 % FBS and granulocyte-colony stimulating factor (G-CSF) (NEUTROGIN®, 100 ng/ml; Chugai Pharmaceutical Co., Ltd, Tokyo, Japan) was added to the 24-well tissue culture plate (BD Biosciences) in the lower chamber. After 48 h of incubation, the medium was changed to DMEM supplemented with 10 % FBS without G-CSF. The MDPSCs by the G-CSF gradient were harvested by 0.05 % Trypsin–EDTA (Life Technologies) prior to 60 % confluency and replated at a 1:4 dilution. The culture medium was switched to DMEM without serum at 50 % confluency at the fourth to fifth passage of culture. The CM from MDPSCs was collected 24 h later and concentrated ~25-fold using an ultrafiltration unit (Amicon Ultra-15 Centrifugal Filter Unit) with a 3-kDa molecular weight cutoff (Ultracel-3 membrane; Millipore, Billerica, MA, USA) and stored with proteinase inhibitors (Halt™ proteinase inhibitor cocktail EDTA-free; Thermo Scientific, Rockford, IL, USA) at –80 °C until use. The protein concentration of the CM was determined by BradfordUltra™ (Expedeon, Cambridge, UK).

### Sequential extraction of induction of microenvironment activity

The tooth roots of porcine second incisors at 1 year of age were dissected to 6 mm in length and 2 mm in width, and were treated by the following process at 4 °C. The periodontal ligament was removed from the surface of the root by an excavator (nonextracted tooth). The teeth were demineralized with 0.6 M HCl for 7 days (HCl-extracted tooth), and further extracted with 4 M GdnHCl in 50 mM Tris–HCl, pH 7.4 for 7 days (GdnHCl-extracted tooth) [13]. They were then extracted with 0.5 M EDTA in 50 mM Tris–HCl, pH 7.4 for the next 7 days (EDTA-extracted tooth) [14]. The GdnHCl extracts and the EDTA extracts were concentrated respectively to 4 μg/ml by centrifugation with an Amicon Ultra-15 Centrifugal Filter Unit with an Ultracel-3 membrane (Millipore) and stored at –80 °C until use. After washing four times with phosphate-buffered saline (PBS, pH 7.4), the four distinct types of the tooth (eight teeth, respectively) were kept in PBS at 4 °C until use.

### Pulp regeneration after ectopic transplantation of the variously extracted teeth

One end of each tooth was sealed with zinc phosphate cement (Elite Cement; GC, Tokyo, Japan). The regenerative potential of each tooth was examined in an in-vivo ectopic tooth transplantation assay. MDPSCs (1 × 10^6^ cells) at the fourth to fifth passage from four different individuals were injected into the teeth with collagen TE (Nitta Gelatin, Osaka, Japan). Each of the four distinct teeth was transplanted subcutaneously both as left portion for histological examination and right portion for real-time RT-PCR analysis in 5-week-old severe combined immunodeficiency (SCID) mice (CB17; CLEA, Tokyo, Japan) (*n* = 16 mice, each two teeth per mouse). Each of the four distinct teeth with surrounding tissue was harvested on day 28 for histology (*n* = 4 mice, respectively) and for real-time RT-PCR analysis (*n* = 4 mice, respectively).

The teeth were fixed in 4 % paraformaldehyde (PFA) (Nakarai Tesque, Kyoto, Japan) at 4 °C overnight, demineralized with Kalkitox™ (Wako Pure Chemical Industries, Ltd, Osaka, Japan), and embedded in paraffin wax (Sigma) for histological analysis. The paraffin sections (5 μm in thickness) were stained with hematoxylin and eosin (H & E). Relative amounts of regenerative tissue were analyzed by capturing video images of the histological preparations under binocular microscopy (M 205 FA; Leica, Wetzlar, Germany) in four sections at 150-μm intervals for four teeth, each transplanted with the nontreated tooth and three type-treated teeth. Newly regenerated tissue and the root canal were traced by on-screen image outlines using Leica Application Suite software. The ratio of the regenerated areas to the root canal areas were calculated (*n* = 4, each four distinct teeth). Cell density was analyzed after counterstaining with Hoechst 33342 (1:1000) on a BZ-9000 BIOREVO fluorescence microscope (KEYENCE, Osaka, Japan). The numbers of Hoechst-positive cells in the regenerated area on day 28 were calculated in three sections of each tooth (*n* = 4 teeth).

Immunohistological analyses with mouse anti-rat rat endothelial cell antigen 1 (RECA1, 1:500; Sanbio BV, Uden, the Netherlands) with biotinylated horse anti-mouse Texas Red secondary antibody (1:200; Vector Lab., Burlingame, CA, USA) were performed to determine the level of neovascularization. The ratio of the area of RECA1-positive newly formed capillaries to the regenerated area on day 28 was calculated in three sections of each tooth (*n* = 4).

In-situ hybridization was performed in the regenerated tissues on day 28 using a marker for pulp, thyrotropin-releasing hormone degrading enzyme (*TRH-DE*), to identify pulp tissue regeneration as described previously [15]. The sections were examined by confocal laser microscopy (TCS SP5 conventional inverted microscope; Leica), and three-dimensional structures were reconstructed by Leica Application Suite Advanced Fluorescence (LAS AF) software (Leica). Normal pulp tissue from the incisors of the SCID mice was used as a positive control (*n* = 4 teeth). Real-time RT-PCR analyses were further performed using *TRH-DE* in the regenerated tissues from each four distinct teeth 28 days after transplantation (*n* = 4).

Odontoblastic differentiation was assessed by in-situ hybridization using a marker for odontoblasts, *enamelysin*, as described previously [6]. DIG signals were detected using a TSA system. The numbers of *enamelysin-*positive cells along the dentinal wall in the regenerated tissue on day 28 were counted in three sections of each tooth (*n* = 4) by LAS AF software using confocal laser microscopy.

### Expression of markers for periodontal ligament in the regenerated tissues

To examine extracellular matrix formation, three paraffin sections of each tooth (*n* = 4) on day 28 were immunostained using rabbit anti-periostin (ab92460, 1:500; abcam, Cambridge, UK) and goat anti-rabbit Alexa 488-conjugated secondary antibody (ab150077, 1:200; abcam) followed by counterstaining with Hoechst 33342. The positive area relative to the regenerated area was analyzed using a BZ9000 BIOREVO fluorescence microscope. The ratio of periostin-positive cells to Hoechst 33342-positive cells in the regenerated tissue on day 28 was calculated in three sections of each tooth (*n* = 4).

Furthermore, immunohistological analysis was performed using a rabbit anti-asporin/periodontal ligament-associated protein 1 (PLAP-1) (ab58741, 1:500; abcam) antibody with a goat anti-rabbit Alexa 488-conjugated secondary antibody (ab150077, 1:200; abcam) followed by counterstaining with Hoechst 33342. The positive area relative to the regenerated area was analyzed using a BZ9000 BIOREVO fluorescence microscope. The ratio of PLAP-1-positive cells to Hoechst 33342-positive cells in the regenerated tissue on day 28 was calculated in three sections of each tooth (*n* = 4).

### Pulp regeneration after ectopic transplantation of the reconstituted teeth with various extracts and CM from MDPSCs

Next, the optimal chemical components of inductive microenvironment for pulp regeneration were assessed. Nonextracted teeth were autoclaved at 121 °C, 2 atm in distilled water to inactivate the chemical components of microenvironment completely and preserve only the physical microenvironment. Some samples of the autoclave teeth were prepared for SEM examination and analyzed using a JXA-8530 F (JEOL, Aichi, Japan). The teeth were then soaked with the GdnHCl extracts or with the EDTA extracts with or without the CM from MDPSCs and were lyophilized at –60 °C, 10 mmHg by Freeze Dryer 8 (Laboconco, Kansas City, KS, USA) for 4 h. Each of four distinct reconstituted teeth were transplanted and harvested for histology (*n* = 4) and for real-time RT-PCR analysis on day 28 (*n* = 4) as already described.

### Proliferation, migration, and cell adhesion assays

The combinatorial effects of the MDPSC CM with the EDTA extracts on enhanced proliferation, migration, and cell attachment were examined to induce higher potential of the CM with the EDTA extracts for pulp and dentin regeneration. For the proliferation assay, after culture without serum overnight, mouse embryonic muscle myoblast cells (C2C12 cells; DS Pharma Biomedical Osaka, Japan) were cultured in DMEM supplemented with 0.2 % BSA, supplemented with the GdnHCl extracts or the EDTA extracts with or without the CM at a final concentration of 5 μg/ml protein. Cell counting kit-8® (Dojindo Molecular Technologies, Kumamoto, Japan) were added to the 96-well plate, and cell numbers were measured using a spectrophotometer at 450 nm absorbance at 2, 12, 24, 36, and 48 h of culture. Wells without cells served as negative controls.

For migration, a horizontal chemotaxis assay was performed using TAXIScan-FL® (Effector Cell Institute, Tokyo, Japan) with C2C12 cells to examine the effect of the GdnHCl extracts or the EDTA extracts with or without the CM on cell migration as described previously [16]. The cell fraction was placed into the single hole with which the device was held together with a stainless steel holder, and the following factors were placed into the contra-hole: 1 μl of 5 μg/ml GdnHCl extracts or the EDTA extracts with or without the CM.

For analysis of cell adhesion, the autoclaved teeth as described earlier were divided in half in the sagittal plane and placed on a 24-well plate with the inside facing upward. C2C12 cells were cultured on the dentin in DMEM with 0.2 % BSA supplemented with the EDTA extracts with or without the CM for 48 h to analyze their enhanced ability for cell adhesion. C2C12 cells cultured on the dentin in DMEM with 10 % FBS were used as a positive control. The attached cells were counted after Giemsa staining in the three areas of each tooth (*n* = 4) using binocular microscopic digital images of the three 1.0 mm × 1.0 mm rectangles scanned in a frame.

### Odontoblastic and endothelial differentiation assays

The combinatorial effects of the MDPSC CM with the EDTA extracts on enhanced odontoblastic and endothelial differentiation were examined to induce higher potential of the CM with the EDTA extracts for pulp and dentin regeneration. To analyze the enhanced odontoblastic differentiation, C2C12 cells were cultured in DMEM with 10 % FBS, 50 mg/ml l-ascorbic acid 2-phosphate (Wako Pure Chemical Industries, Ltd), and 1 mM inorganic phosphate (Pi; Sigma-Aldrich) supplemented with the EDTA extracts alone or together with the CM for 28 days. Bone morphogenetic protein (BMP2; kindly provided by Astellas Pharma Co., Ltd, Tokyo, Japan) at a final concentration of 100 ng/ml was used as a positive control. RT-PCR analyses were further performed using primers for odontoblast differentiation markers, dentin sialophosphoprotein (*DSPP*) and *enamelysin* (Table 1), in the cells from each of three dishes (*n* = 3).

**Table 1:** Mouse primers for RT-PCR

Gene		5' ← DNA sequence → 3'	Product size	Accession number
*Enamelysin*	Forward	GGCGAGATGGTGGCAAGA	163 bp	NM_013903
	Reverse	GGAAGAGGCGGTAGTTAG		
*DSPP*	Forward	GGAACTGCAGCACAGAATGA	199 bp	NM_010080
	Reverse	CAGTGTTCCCCTGTTCGTTT		
*β-actin*	Forward	AAGTACCCCATTGAACACGG	257 bp	NM_007393
	Reverse	ATCACAATGCCAGTGGTACG		

*bp* base pair, *DSPP* dentin sialophosphoprotein

To analyze the enhanced endothelial differentiation, human umbilical vein endothelial cells (HUVEC) were cultured in DMEM containing 2 % FBS, 1 μg/ml heparin (Lonza, Muenchensteinerstrasse, Switzerland), 1 μg/ml ascorbic acid (Lonza), and 0.4 μg/ml hydrocortisone (Lonza) supplemented with the EDTA extracts alone or together with the CM for 14 days. Vascular endothelial growth factor (VEGF) (Lonza), basic fibroblast growth factor (b-FGF) (Lonza), and insulin-like growth factor (IGF) (Lonza) at a final concentration of 1 μg/ml, respectively, was used as a positive control. Immunocytochemical analyses were performed for anti-vascular endothelial (VE)-cadherin (primary antibody, 1:50; Acris, Herford, Germany), and the positive cells were observed on a BZ-9000 BIOREVO fluorescence microscope after counterstaining with Hoechst 33342.

### Statistical analyses

Data are reported as means ± SD. *P* values were calculated using the Student’s *t* test and Tukey’s multiple comparison test in SPSS 21.0 (IBM, Armonk, NY, USA).

## Results

### Pulp/dentin regeneration after tooth transplantation

**Fig. 1:**
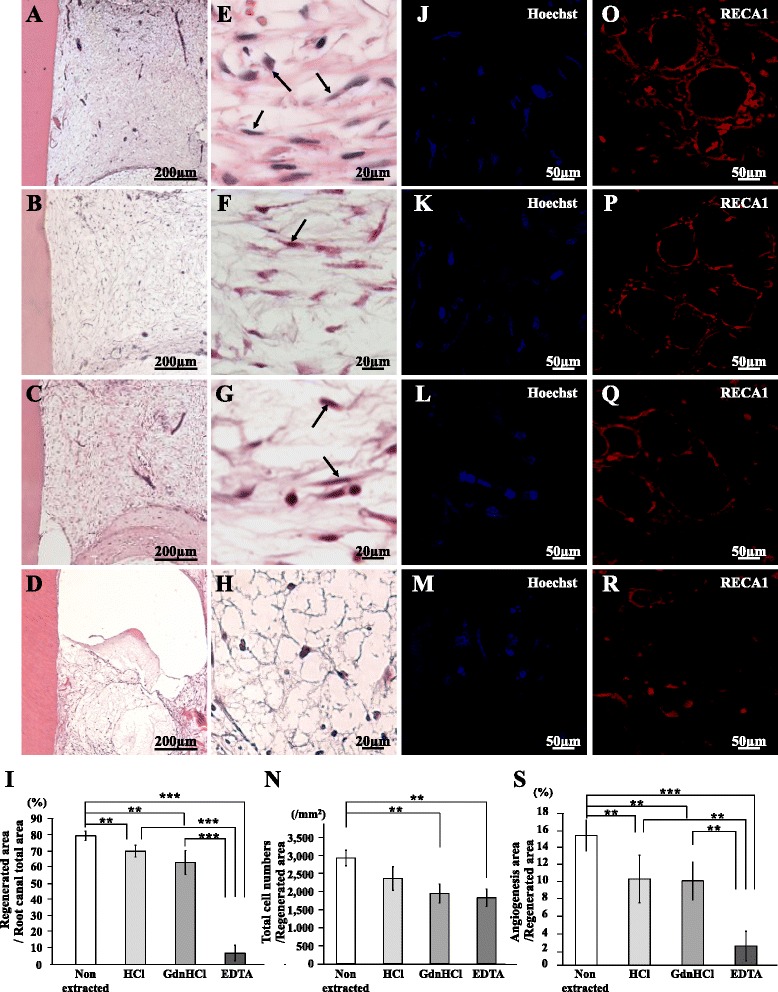
Pulp regeneration after ectopic tooth root transplantation. Pulp regeneration after ectopic tooth root transplantation in SCID mice. Twenty-eight days after transplantation of MDPSCs with (**a**, **e**, **j**, **o**) nonextracted tooth, (**b**, **f**, **k**, **p**) HCl-extracted tooth, (**c**, **g**, **l**, **q**) GdnHCl-extracted tooth, and (**d**, **h**, **m**, **r**) EDTA-extracted tooth. **a**–**h** H & E staining. Pulp-like cells (*arrows*). **i** Ratio of regenerated area to root canal area. **j**–**m** Hoechst 33342 staining. **n** Ratio of Hoechst 33342-positive cells to regenerated area. **o**–**r** Immunostaining with rat endothelial cell antigen 1 (*RECA1*). **s** Ratio of vascularization area to the total regenerated area. Data are expressed as means ± SD of four determinations. **P < 0.01, ***P < 0.001

The regenerative potential of the three distinct types of extracted teeth was compared with control nonextracted tooth in an ectopic tooth transplantation assay of SCID mice. Pulp-like tissue with well-organized vasculature was regenerated in the teeth 28 days after MDPSC transplantation as a positive control (Fig. 1a, e). Similar pulp-like loose connective tissue was observed in the transplants of the teeth extracted with HCl, GdnHCl, and EDTA (Fig. 1b–d, f–h) and in the transplant of nonextracted teeth (Fig. 1a, e). The regenerated tissue in the EDTA-extracted tooth transplant (Fig. 1m) had fewer Hoechst 33342-stained cells compared with those in the nonextracted, HCl-extracted, and GdnHCl-extracted tooth transplants (Fig. 1j–l). The histomorphometric analysis confirmed that the regenerated pulp area and cell density of the GdnHCl-extracted tooth transplants and the EDTA-extracted tooth transplants were significantly lower than those of the nonextracted tooth transplants on day 28 (Fig. 1n). The histomorphometric analysis confirmed that the regenerated pulp area in the tooth transplants of the three types of treatment was significantly lower than that of the nontreatment on day 28 (Fig. 1i). There were no significant differences in the regenerated area between the HCl-extracted tooth transplant and the GdnHCl-extracted tooth transplant. Transplantation of the EDTA-extracted teeth yielded significantly less regenerated tissue compared with those of the other three teeth on day 28 (Fig. 1i). These results suggest that chemical components extracted by EDTA may mainly generate an inductive microenvironment for pulp regeneration. Immunostaining with a RECA1 antibody revealed neovascularization in the regenerated tissues by nonextracted tooth transplantation and the other three types of tooth transplantation (Fig. 1o–r). Histomorphometric analysis demonstrated that neovascularization in the nonextracted tooth transplant was significantly higher than that in the HCl-extracted, GdnHCl-extracted, and EDTA-extracted tooth transplants on day 28. There was no significant difference in neovascularization between the HCl-extracted and GdnHCl-extracted tooth transplants, and a significant difference between the EDTA-extracted tooth transplant and others (Fig. 1s). These results suggest that chemical components extracted by EDTA may mainly generate an inductive microenvironment for pulp regeneration and neovascularization.

**Fig. 2:**
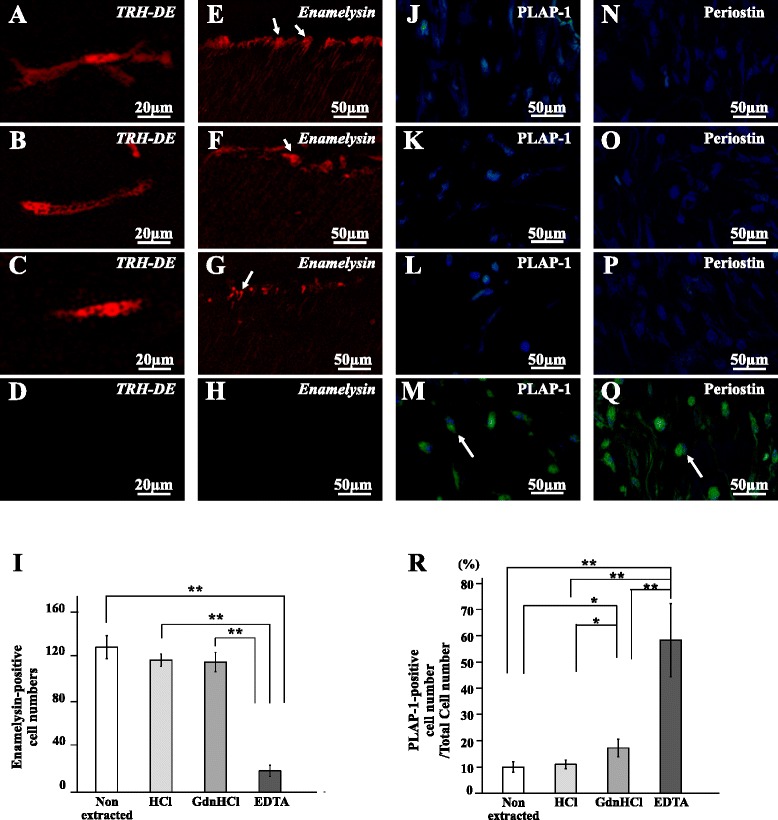
Characterization of regenerated tissue after extracted tooth transplantation. Twenty-eight days after transplantation of (**a**, **e**, **j**, **n**) nonextracted tooth, (**b**, **f**, **k**, **o**) HCl-extracted tooth, (**c**, **g**, **l**, **p**) GdnHCl-extracted tooth, and (**d**, **h**, **m**, **q**) EDTA-extracted tooth. **a**–**d** In-situ hybridization analysis of mRNA expression of thyrotropin-releasing hormone degrading enzyme (*TRH-DE*) as a pulp marker. **e**–**h** In-situ hybridization analysis of *enamelysin* as an odontoblast marker using an anti-sense probe reactive to both porcine and mouse genes. Odontoblastic processes (*arrows*) extending into the tubular dentin. **i** Comparison of the numbers of *enamelysin*-positive cells along the dentinal wall. **j**–**m** Immunostaining with periodontal ligament-associated protein 1 (*PLAP-1*, *green*) merged with Hoechst 33342 (*blue*). **n**–**q** Immunostaining with periostin (*green*) merged with Hoechst 33342 (*blue*). Typical positive cells (*arrows*). **r** Ratio of the PLAP-1-positive cell number to the total cell number. Data are expressed as means ± SD of four determinations. *P < 0.05, **P < 0.01 (Color figure online)

*TRH-DE*, a specific marker for pulp tissue, was similarly expressed in the regenerated tissues of the nonextracted, the HCl-extracted, and the GdnHCl-extracted tooth transplants as analyzed by in-situ hybridization (Fig. 2a–c). However, there were no *TRH-DE*-positive cells in EDTA-extracted tooth transplant (Fig. 2d). Real-time RT-PCR analyses demonstrated similar expression levels of *TRH-DE* mRNA in the regenerated tissue of the nonextracted, HCl-extracted, and GdnHCl-extracted tooth transplants to that in normal pulp tissue, which was significantly higher than that of the EDTA-extracted tooth transplant (Table 2).

**Table 2:** Relative mRNA expression of *TRH-DE* in regenerated tissues of the transplants of nonextracted and extracted teeth compared with normal pulp

	Nonextracted	HCl extracted	GdnHCl extracted	EDTA extracted
*TRH-DE*	1.0 ± 0.1	0.8 ± 0.2	0.9 ± 0.2	0

*EDTA* ethylenediaminetetraacetic acid, *GdnHCl* guanidine hydrochloride, *TRH-DE* thyrotropin-releasing hormone degrading enzyme

*Enamelysin-*positive cells were attached to the dentinal wall, extending their processes for some distance into the dentin in the HCl-extracted and GdnHCl-extracted tooth transplants similar to the nonextracted tooth transplant (Fig. 2e–g), and they were few in the EDTA extracted tooth transplant (Fig. 2h). The quantitative analysis demonstrated that the number of *enamelysin*-positive cells was significantly higher in the regenerated tissues in the nonextracted, HCl-extracted, and GdnHCl-extracted tooth transplants compared with that of the EDTA-treated tooth transplant (Fig. 2i). Furthermore, immunohistochemical analyses demonstrated minimal expression of PLAP-1 and periostin, the markers for periodontal ligament in the regenerated tissues by the nonextracted, HCl-extracted, and GdnHCl-extracted tooth transplantation (Fig. 2j–q). The quantitative analysis indicated that the number of PLAP-1-positive cells was significantly lower in the regenerated tissues of the nonextracted, HCl-extracted, and GdnHCl-extracted tooth transplants compared with that in the EDTA-extracted tooth transplant (Fig. 2r). These results suggest that the EDTA extracts may contain potential chemical components of an inductive microenvironment for dentin regeneration.

### Pulp/dentin regeneration after reconstitution of soluble extracts with the autoclaved teeth

**Fig. 3:**
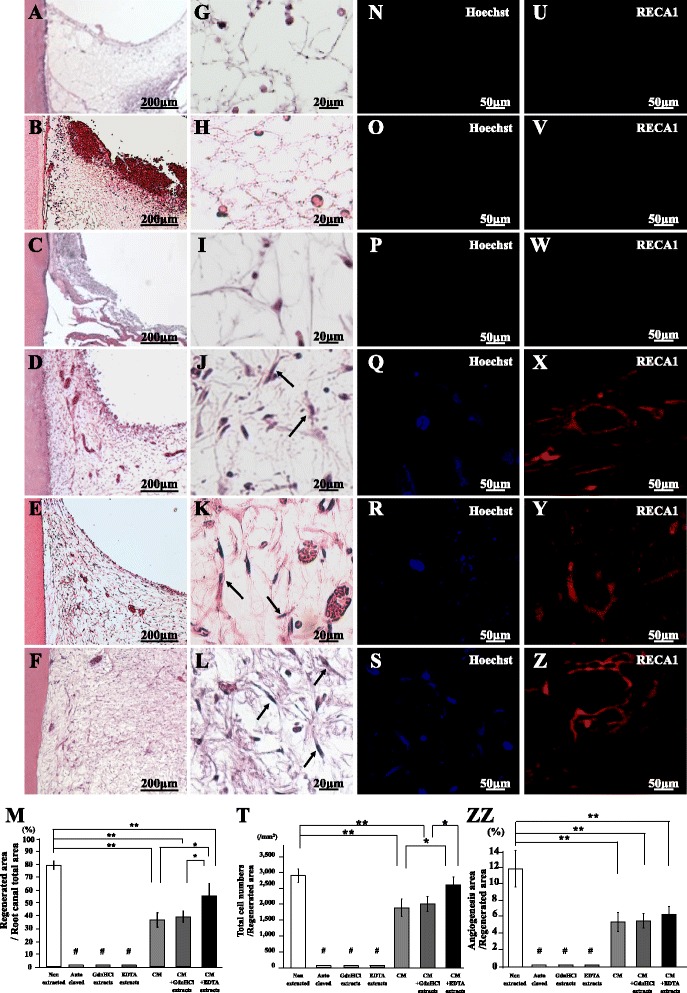
Pulp regeneration after transplantation of the autoclaved teeth reconstituted with soluble extracts. Twenty-eight days after ectopic transplantation of MDPSCs (**a**, **g**, **n**, **u**) in the native autoclaved teeth only, and in the autoclaved teeth reconstituted (**b**, **h**, **o**, **v**) with the GdnHCl extracts, (**c**, **i**, **p**, **w**) with the EDTA extracts, (**d**, **j**, **q**, **x**) with the MDPSC CM, (**e**, **k**, **r**, **y**) with the GdnHCl extracts and the CM, and (**f**, **l**, **s**, **z**) with the EDTA extracts and the CM. **a**–**l** H & E staining. Pulp-like cells (*arrows*). **m** Ratio of regenerated area to root canal area. **n**–**s** Hoechst 33342 staining. **t** Ratio of Hoechst 33342-positive cells to regenerated area. **u**–**z** Immunostaining with RECA1. **zz** Ratio of vascularization area to the total regenerated area. Data are expressed as means ± SD of four determinations. *P < 0.05, **P < 0.01, ^#^ *P* < 0.001 to nonextracted tooth, reconstituted tooth with the MDPSC CM alone, and MDPSC CM with the GdnHCl extracts or the EDTA extracts. *CM* conditioned medium, *RECA1* rat endothelial cell antigen 1

**Fig. 4:**
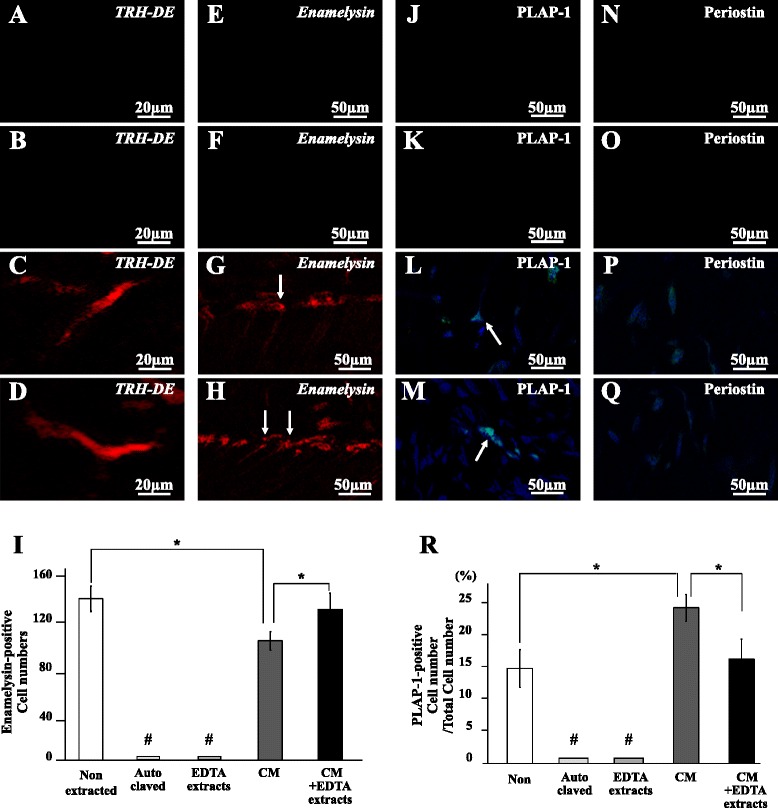
Characterization of regenerated tissues after transplantation of reconstituted teeth. Twenty-eight days after ectopic transplantation of MDPSCs (**a**, **e**, **j**, **n**) in the native autoclaved tooth, in the autoclaved teeth reconstituted (**b**, **f**, **k**, **o**) with the EDTA extracts, (**c**, **g**, **l**, **p**) with the CM, and (**d**, **h**, **m**, **q**) with the EDTA extracts and the CM. **a**–**d** In-situ hybridization analysis of expression of thyrotropin-releasing hormone degrading enzyme (*TRH-DE*) as a pulp marker. **e**–**h** In-situ hybridization analysis of *enamelysin*. Odontoblastic processes (*arrows*) extending into the tubular dentin. **i** Comparison of the numbers of *enamelysin*-positive cells along the dentinal wall. **j**–**m** Immunostaining with periodontal ligament-associated protein 1 (*PLAP-1*, *green*) merged with Hoechst 33342 (*blue*). A typical PLAP-1-positive cell (*arrow*). **n**–**q** Immunostaining with periostin (*green*) merged with Hoechst 33342 (*blue*). **r** Ratio of the PLAP-1-positive cell number to the total cell number. Data are expressed as means ± SD of four determinations. *P < 0.05. ^#^ *P* < 0.001 to nonextracted tooth, reconstituted tooth with the MDPSC CM alone, and the MDPSC CM with the EDTA extracts. *CM* conditioned medium (Color figure online)

To examine the chemical components of inductive microenvironment, the regenerative potential of the reconstituted autoclaved teeth with the EDTA extracts with or without the MDPSC CM was examined in the ectopic tooth transplantation model. The autoclaved teeth similarly preserved the tubules as nonautoclaved teeth analyzed by SEM, indicating intact physical microenvironment (Additional file 1: Figure S1). The reconstituted autoclaved teeth with the GdnHCl extracts with or without the CM were used as a control for the EDTA extracts. Pulp-like tissue was not regenerated in the autoclaved teeth reconstituted with the GdnHCl extracts or the EDTA extracts and in the autoclaved tooth without any reconstitution 28 days after transplantation (Fig. 3a–c, g–i). On the other hand, pulp-like tissue with well-organized vasculature was observed in the autoclaved tooth reconstituted with the CM (Fig. 3d, j). Furthermore, the autoclaved tooth reconstituted with both the EDTA extracts and the CM (Fig. 3f, l) demonstrated a higher amount of regenerated tissue compared with that with the EDTA extracts alone (Fig. 3c, i) or the CM alone (Fig. 3d, j). The histomorphometric analysis confirmed that reconstitution of the EDTA extracts together with the CM significantly enhanced the regenerated area compared with either alone in the autoclaved tooth (Fig. 3m). The combinatorial effect of the EDTA extracts on the pulp regeneration with the CM was detected, unlike the GdnHCl extracts (Fig. 3m). No cell was stained with Hoechst 33342 nor RECA1 in the autoclaved tooth and the reconstituted tooth of the EDTA extracts as well as that of the GdnHCl extracts (Fig. 3n–p, u–w). The reconstitution with the EDTA extracts together with the CM increased the number of Hoechst-positive cells in the regenerated tissue (Fig. 3s) compared with the reconstitution with the CM alone (Fig. 3q). The histomorphometric analysis confirmed that the regenerated pulp area and cell density of the transplants of the tooth reconstituted with the CM alone or the tooth reconstituted with the GdnHCl extracts and the CM were significantly lower than those of the transplant of nontreated tooth on day 28. On the other hand, those of the tooth transplant reconstituted both with the EDTA extracts and the CM were significantly higher compared with the tooth transplant reconstituted with the CM or the tooth transplant reconstituted with the GdnHCl extracts and the CM (Fig. 3m, t). Immunostaining with an RECA1 antibody revealed neovascularization in the regenerated tissue of the reconstituted tooth transplants of the CM with or without the EDTA extracts (Fig. 3x, z). Histomorphometric analysis demonstrated that there were no significant differences in the neovascularization between teeth reconstituted with the CM and with the CM with the EDTA extracts (Fig. 3zz). There were no *TRH-DE*-positive cells in the autoclaved tooth reconstituted with or without the EDTA extracts as analyzed by in-situ hybridization (Fig. 4a, b). However, *TRH-DE* was similarly expressed in the transplants of autoclaved teeth reconstituted with the CM alone and with the CM and the EDTA extracts together (Fig. 4c, d). The real-time RT-PCR analyses demonstrated that *TRH-DE* was similarly expressed in the regenerated tissue in the transplants of the teeth reconstituted with the CM alone or with the CM and the EDTA extracts as in normal pulp tissue (Table 3), suggesting that the regenerated tissue in these reconstituted teeth may be pulp tissue.

**Table 3:** Relative mRNA expression of *TRH-DE* in regenerated tissues of the transplants of reconstituted autoclaved teeth with the EDTA extracts, the CM alone, and the EDTA extracts with the CM compared with normal pulp

	EDTA extracts	CM	CM + EDTA extracts
*TRH-DE*	0	0.8 ± 0.3	1.0 ± 0.3

*CM* conditioned medium, *EDTA* ethylenediaminetetraacetic acid, *TRH-DE* thyrotropin-releasing hormone degrading enzyme

There were no *enamelysin-*positive cells in the autoclaved tooth and the reconstituted tooth of the EDTA extracts (Fig. 4e, f). On the other hand, there were *enamelysin-*positive cells in the autoclaved tooth with the CM and with the CM and the EDTA extracts. Statistical analysis showed that the number of *enamelysin-*positive cells was significantly higher in the autoclaved tooth reconstituted with the CM and the EDTA extracts compared with that in the CM alone reconstituted (Fig. 4i). On the other hand, there were no significant differences in the number of *enamelysin-*positive cells between nontreated tooth and reconstituted autoclaved tooth. Furthermore, immunohistochemical analyses of PLAP-1 and periostin, markers for periodontal ligament, demonstrated a positive response in the regenerative tissue in the reconstituted teeth with the CM or with the CM and the EDTA extracts (Fig. 4l, m). Quantitative analysis indicated that the number of PLAP-1-positive cells was significantly higher in the regenerated tissue of the CM reconstituted tooth transplant compared with that in the nonextracted tooth and autoclaved tooth reconstituted with the CM and the EDTA extracts (Fig. 4r). These results demonstrated that the EDTA extracts may enhance the inductive effect of the CM on odontoblastic differentiation.

### Influence of the EDTA extracts and the MDPSCs CM in vitro

**Fig. 5:**
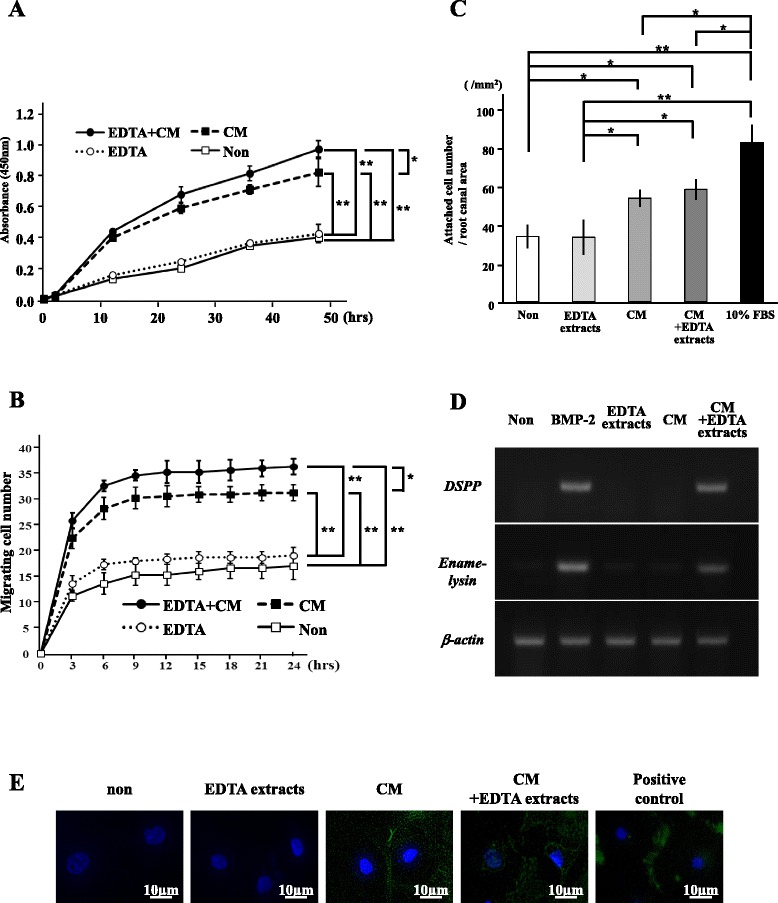
Effects of the EDTA extracts and the CM on proliferation, migration, cell attachment, odontoblastic differentiation, and endothelial cell differentiation in vitro. **a** Proliferation effect of the EDTA extracts and the CM analyzed by CCK-8® in C2C12 cells. **b** Migration effect of the EDTA extracts and the CM analyzed by TAXIScan-FL® in C2C12 cells. **c** Effect of the EDTA extracts and the CM on cell attachment ability analyzed by Giemza stain in C2C12 cells. **d** Effect of the EDTA extracts and the CM on odontoblast differentiation analyzed by RT-PCR in C2C12 cells. **e** Angiogenic effect of the EDTA extracts and the CM by immunocytochemical analysis with vascular endothelial (VE)-cadherin in HUVEC. Data are expressed as means ± SD of four determinations. *P < 0.05, **P < 0.01. *CM* conditioned medium, *DSPP* dentin sialophosphoprotein

During the process of pulp/dentin regeneration, it is well known that the cells surrounding the tooth migrate into the tooth canal, adhere/engraft to scaffold/microenvironment, and proliferate and differentiate into pulp cells/odontoblasts. Thus, to analyze the in-vivo effects of the EDTA extracts on pulp/dentin regeneration, the effects of the EDTA extracts on proliferation, migration, cell attachment, and odontoblastic differentiation were examined in vitro combined with or without the MDPSC CM. The proliferation analysis demonstrated that the MDPSC CM had a significantly higher effect on enhanced proliferation than nontreatment (Fig. 5a). However, there were no significant differences between nontreatment and EDTA extracts. Furthermore, the CM with the EDTA extracts showed a significantly higher proliferation effect compared with the CM alone. The EDTA extracts or the CM significantly increased the migration activity of C2C12 cells. The EDTA extracts with the CM presented significantly higher migration activity than the EDTA extracts alone or the CM alone (Fig. 5b). The CM also had significantly higher cell attachment ability than nontreatment and the EDTA extracts. Furthermore, the EDTA extracts showed no significant increase in cell attachment ability of the CM (Fig. 5c), suggesting no effect of the EDTA extracts on cell adhesion. The RT-PCR analysis demonstrated that the EDTA extracts together with the CM induced odontoblastic differentiation, although those alone had no effect (Fig. 5d). The angiogenic potential was demonstrated by immunostaining with VE-cadherin on day 14. The CM induced differentiation of HUVEC into an endothelial cell lineage positive for VE-cadherin. However, the EDTA extracts showed no increase in cell endothelial differentiation (Fig. 5e).

## Discussion

The long-term goal of tissue engineering in dentistry is the design and optimization of new functional teeth with dentin and pulp. The realization of this goal will overcome the limitations of artificial dental prosthesis. The critical challenge in tissue engineering is to optimize stem cells, morphogens to direct stem cells in the inductive microenvironment of extracellular matrix (ECM) scaffold.

The development of novel approaches to control the lineage of stem cell fate is a prerequisite for tissue engineering and regenerative medicine. The three-dimensional microenvironment has a profound influence on the proliferation, differentiation, and maintenance of MSCs [17]. The inductive microenvironment consists of physical cues of ECM stiffness and the topology of ECM and instructive biochemical signals in the form of soluble or ECM-bound morphogens, growth factors, and cytokines [9, 18–21]. The three-dimensional biomimetic silicon scaffold with dentinal tubule-like pones is a prerequisite for odontoblastic differentiation of MSCs [22].

In this present investigation, the biomimetic tooth ECM scaffold for dentin and dental pulp was investigated systematically. The methodological approach consisted of sequential extraction of porcine incisor teeth by 0.6 M HCl to demineralize, and extraction in 4 M GdnHCl, pH 7.4 and 0.5 M EDTA, pH 7.4. The extracted tooth matrix was combined with MDPSC CM, and was evaluated in SCID mice for pulp/dentin regeneration. In addition, the extracted ECM components obtained by the GdnHCl extracts and the EDTA extracts (with or without MDPSC CM) were reconstituted with native (nonextracted) teeth that were autoclaved to inactivate the endogenous bioactive components. The reconstituted teeth were transplanted into an ectopic site in recipient SCID mice to unambiguously evaluate the GdnHCl extracts and the EDTA extracts. The 0.5 M EDTA extraction reduced the inductive potential for pulp regeneration, odontoblastic differentiation, and angiogenesis/vasculogenesis in the tooth transplants. Whereas the periodontal marker PLAP-1 was expressed, the pulp marker *TRH-DE* was not detectable. A recent report has demonstrated that the EDTA treatment of the root surface could help to expose collagen fibers on root cementum and enhance the surface roughness, promoting the adherence cytokines and the release of inherent proteins or growth factors within the tooth [23]. This report demonstrated regeneration for periodontal ligament and root surface. However, the effects of EDTA treatment for pulp regeneration are not well understood. These findings suggest that 0.5 M EDTA may extract select biochemical components involved in inductive microenvironment for pulp regeneration. It is well known that the EDTA extracts are enriched in noncollagenous proteins [24] including, but not limited to, osteonectin, osteocalcin, osteopontin, bone sialoprotein, and DSPP [25, 26]. DSPP is cleaved by a protease to dentin sialoprotein (DSP), dentin glycoprotein (DGP), and dentin phosphoprotein (DPP). ECM comprises enzymes and matrix-bound growth factors as well as intrinsic ECM components. The ECM-bound growth factors may regulate growth factor activity and cellular proliferation and differentiation [27, 28]. The EDTA extracts may therefore contain a constellation of noncollagenous proteins and the EDTA-bound growth factors.

Based on the finding of bioactive factors in EDTA extracts we next examined the GdnHCl extracts and the EDTA extracts reconstituted with biologically inactivated teeth obtained by autoclaving that preserves physical cues of the pores of the dentinal tubules. However, both the GdnHCl extracts and the EDTA extracts were devoid of pulp/dentin regenerative potential. It is noteworthy that the CM from the MDPSCs reconstituted with autoclaved teeth demonstrated copious amounts of regenerated pulp tissue. In an unexpected discovery, the addition of the EDTA extracts to the MDPSC CM significantly increased pulp tissue compared with the MDPSC CM alone. The attachment of MDPSCs to the dentinal wall of the autoclaved teeth is a prerequisite for pulp regeneration. The MDPSC CM contains soluble factors including BDNF, EGF, FGF2, GDNF, GM-CSF, HGF, MMP3, NPY, NGF, PDGF, TGFβ, and VEGF [29]. The earlier work demonstrated that the presence of multiple growth factors in the MDPSC CM was higher compared with that in the MBMSC CM and the MADSC CM. The observed higher regenerative potential of MDPSC CM may be due to higher secretion of trophic factors for angiogenesis, migration, and anti-apoptosis compared with BMSCs and ADSCs [6, 30].

The chemical cues in the EDTA extract may induce odontoblast differentiation. The candidate molecules may include DSP, DGP, and DPP [27]. DSP has an inductive potential for mineralization in vitro [31] and has a role in initiation of dentin mineralization and maturation of odontoblasts [32, 33]. The MDPSC CM also induced odontoblast differentiation in vitro [34]. It should be noted that the DPSC CM contains BMP4, BMP7, and Wnt10a [22, 35]. As demonstrated in this investigation, the autoclaved tooth transplantation demonstrated no pulp regeneration and no odontoblast differentiation. However, combination of the EDTA extracts with the MDPSC CM with autoclaved teeth demonstrated significant pulp regeneration and odontoblast differentiation. The combinatorial effects on proliferation, migration, and odontoblast differentiation were also demonstrated in vitro. Thus, when transplanted with autoclaved teeth, there is collaboration between the physical cues in the microenvironment of the autoclaved teeth and the potent biochemical cues present in the EDTA extracts and the MDPSC CM. The detailed future investigations of the collaborative interactions between the physical cues of the ECM and chemical cues in the EDTA extracts of the tooth ECM will inform and enhance the platform of tissue engineering in dentistry and oral regenerative medicine.

## Conclusions

The EDTA-extracted teeth demonstrated significantly lower pulp/dentin regenerative potential compared with the GdnHCl-extracted teeth. The EDTA soluble chemical components and the MDPSC CM, when reconstituted with the physical structure of autoclaved teeth, served as an inductive microenvironment, promoting cell proliferation, migration, and odontoblastic differentiation.

## Abbreviations

CM, conditioned medium; DMEM, Dulbecco’s modified Eagle’s medium; ECM, extracellular matrix; EDTA, ethylenediaminetetraacetic acid; FBS, fetal bovine serum; G-CSF, granulocyte-colony stimulating factor; GdnHCl, guanidine hydrochloride; HCl, hydrochloric acid; H & E, hematoxylin and eosin; HUVEC, human umbilical vein endothelial cells; MDPSC, mobilized dental pulp stem cell; MSC, Mesenchymal stem cell; PBS, phosphate-buffered saline; TRH-DE, thyrotropin-releasing hormone degrading enzyme; VE-cadherin, vascular endothelial cadherin.

## Additional file

Additional file 1: Figure S1. Showing the effect of autoclave treatment on the physical microenvironment of the dentin surface. SEM images demonstrating **A**, **B** nontreated dentin surface and **C**, **D** autoclaved dentin surface. (PDF 227 kb)
