# Supplementary figures and images for: EDTA soluble chemical components and the conditioned medium from mobilized dental pulp stem cells contain an inductive microenvironment, promoting cell proliferation, migration, and odontoblastic differentiation
Source: Stem Cell Res Ther. 2016 May 25;7:77. doi: 10.1186/s13287-016-0334-z (PMC4937592; doi:10.1186/s13287-016-0334-z)

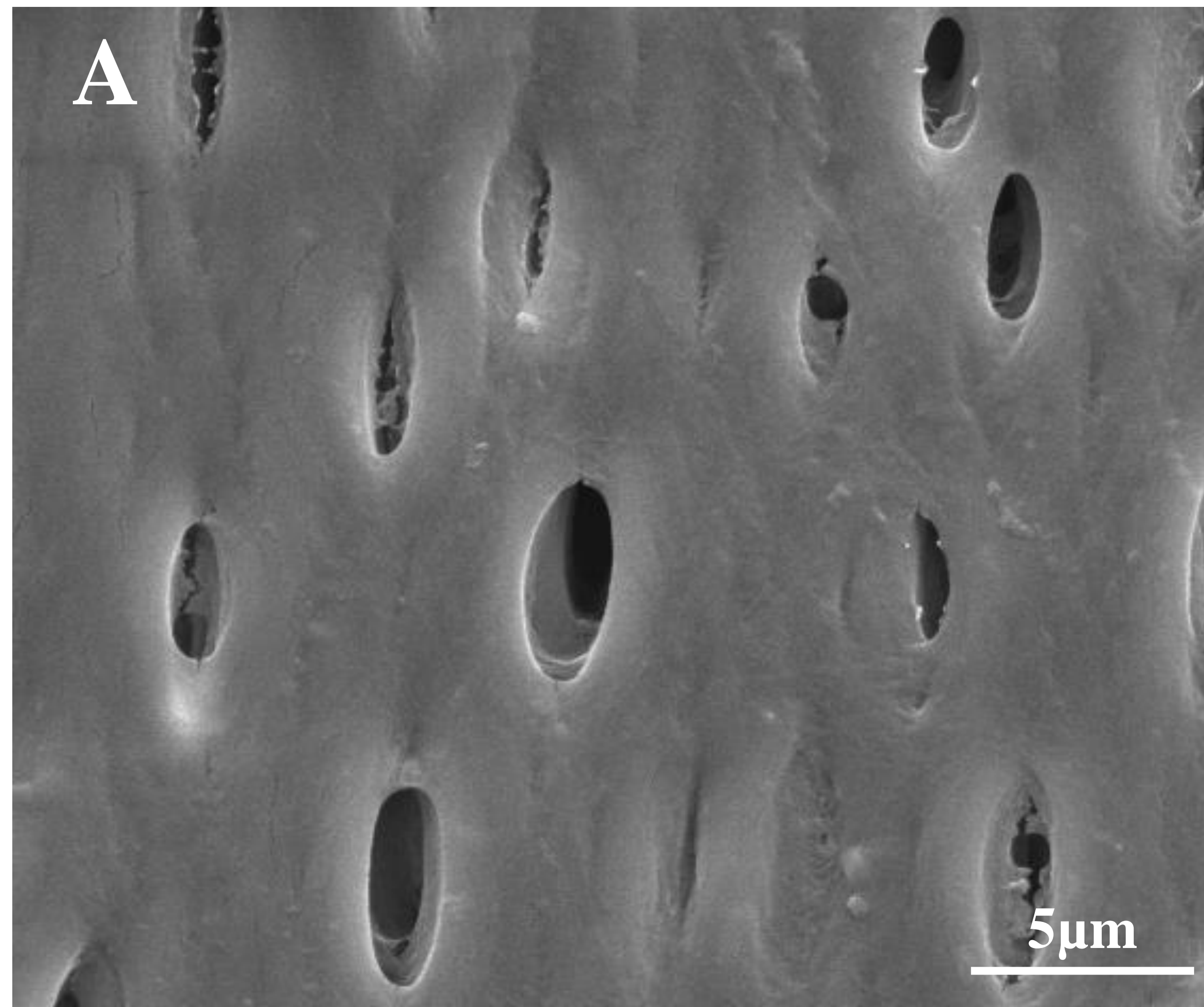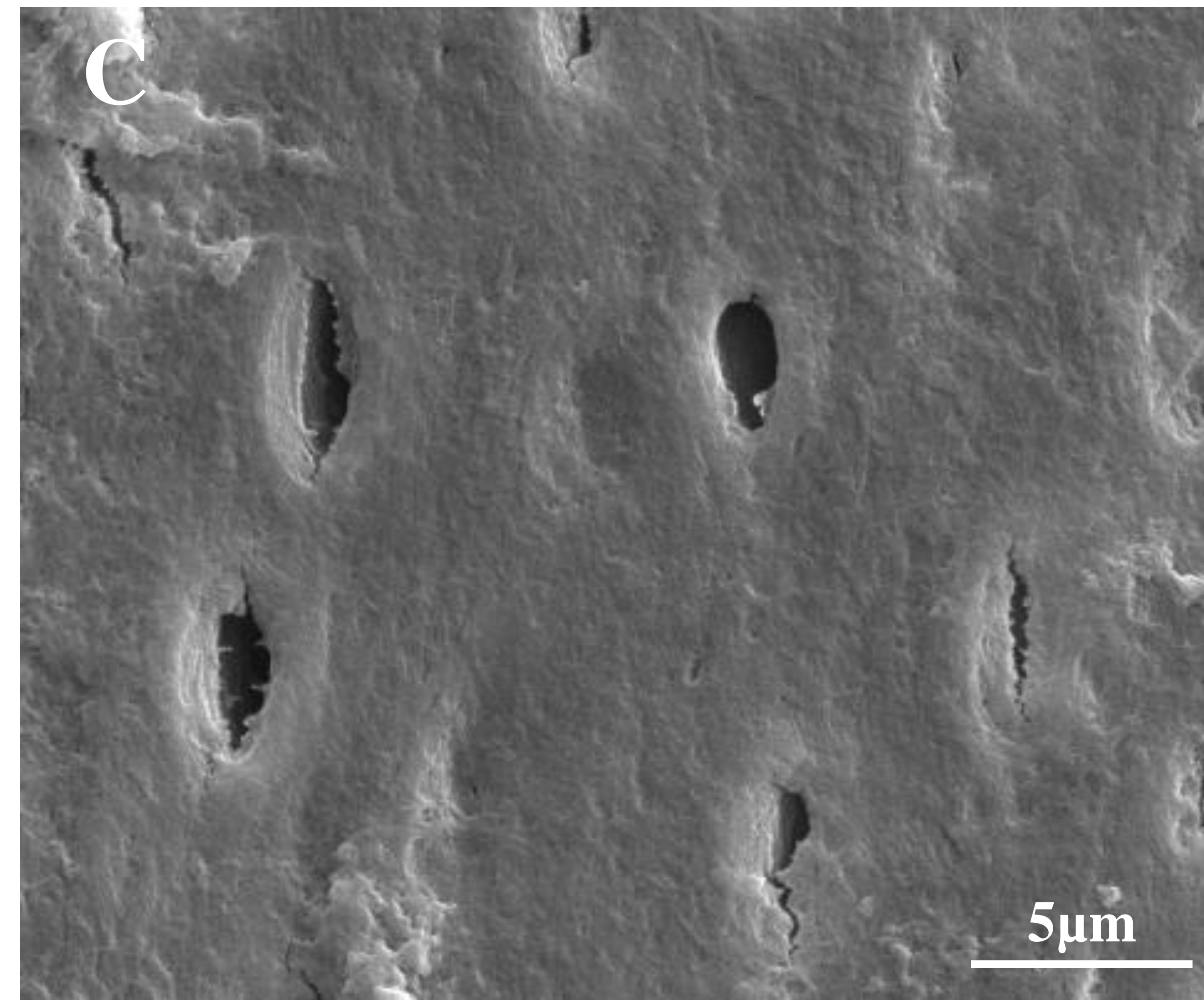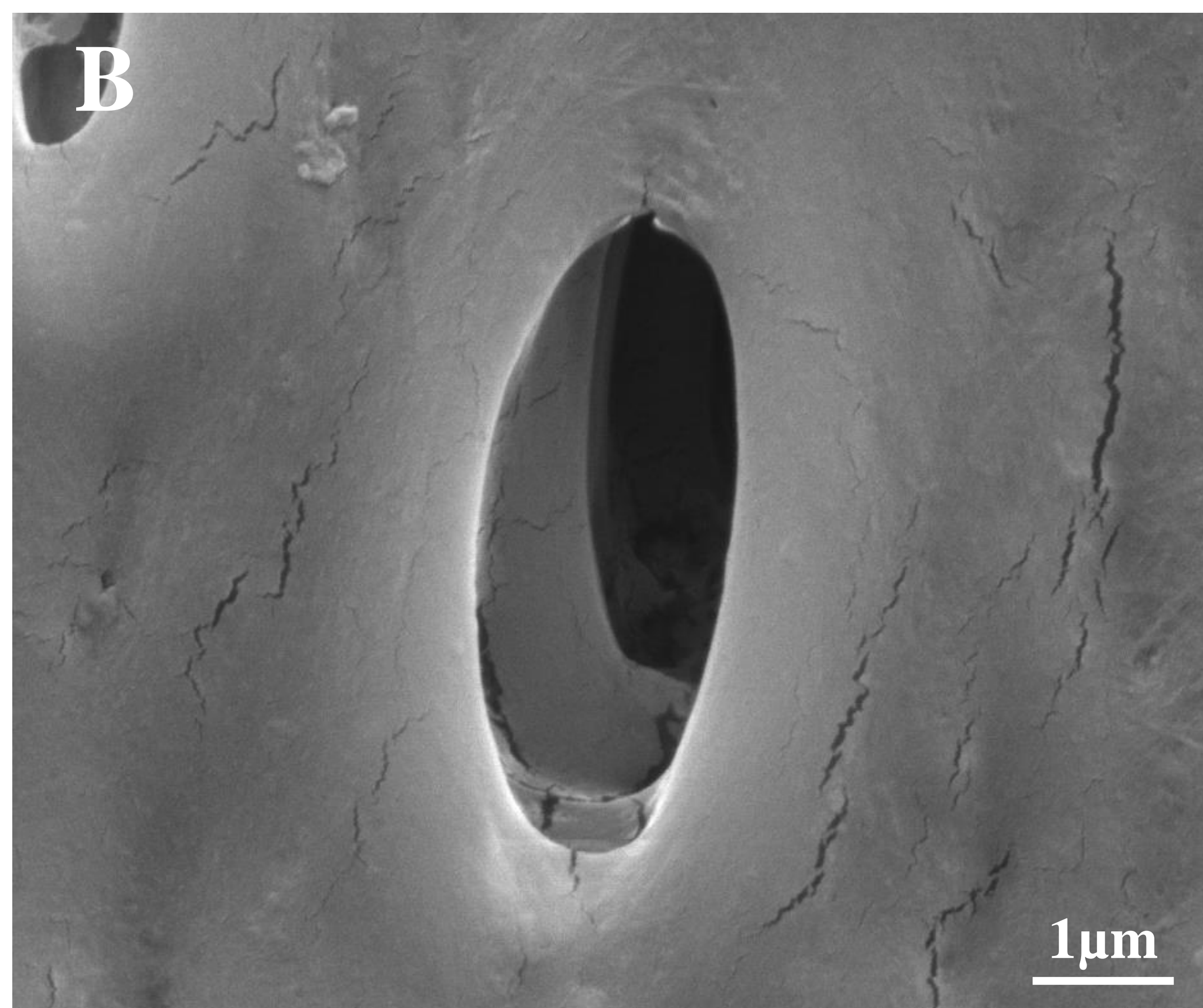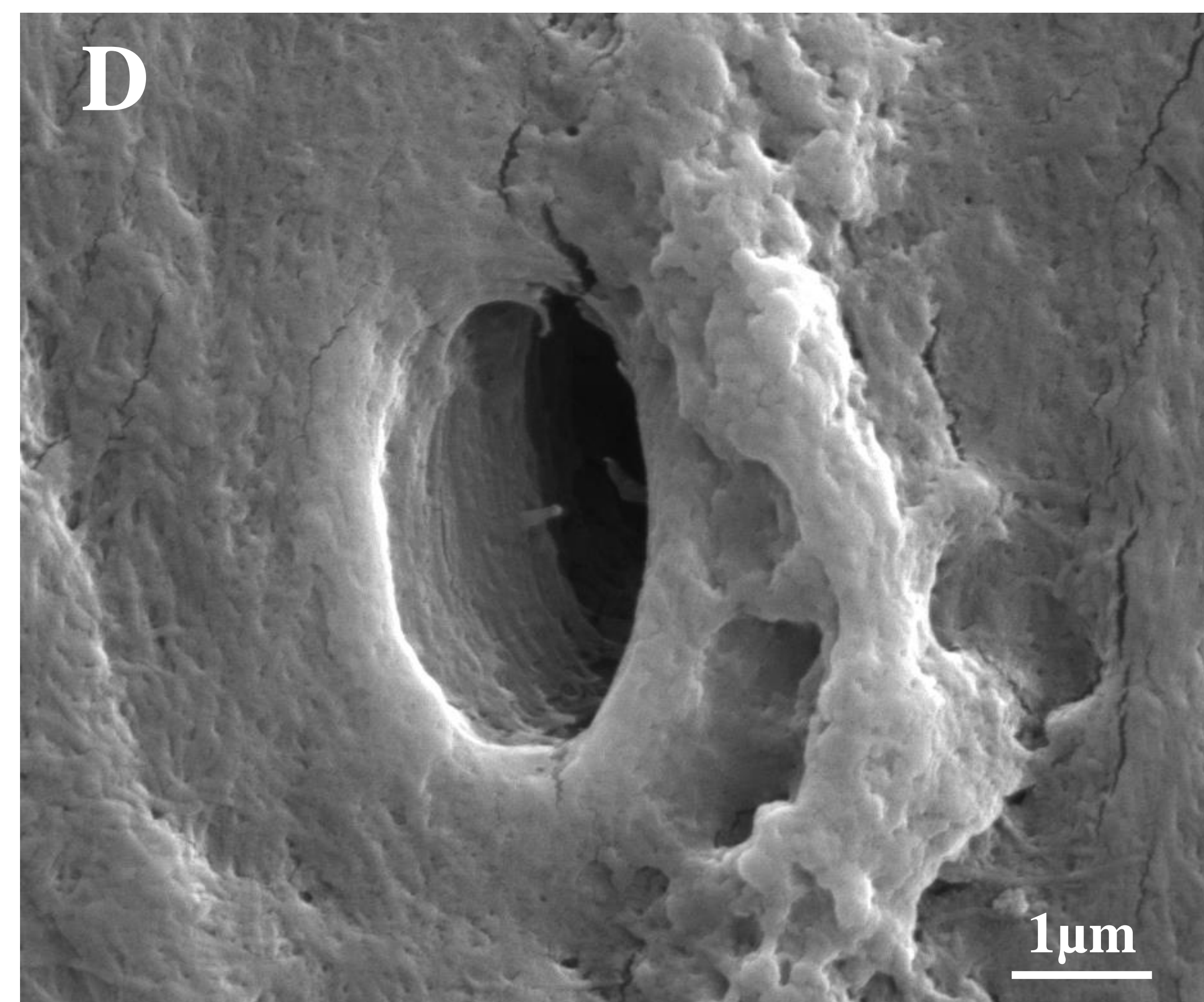

Supplemental  
Fig. 1

Supplement: Additional file 1: Figure S1. — Showing the effect of autoclave treatment on the physical microenvironment of the dentin surface. SEM images demonstrating A, B nontreated dentin surface and C, D autoclaved dentin surface. (PDF 227 kb) [file 13287_2016_334_MOESM1_ESM.pdf]
